# Sexual Orientation, Drug Use Preference during Sex, and HIV Risk Practices and Preferences among Men Who Specifically Seek Unprotected Sex Partners via the Internet

**DOI:** 10.3390/ijerph6051620

**Published:** 2009-05-11

**Authors:** Hugh Klein

**Affiliations:** Kensington Research Institute, 401 Schuyler Road, Silver Spring, Maryland, 20910, USA; Center for the Study and Prevention of Drug Use, Morgan State University, Baltimore, Maryland, USA; E-Mail: hughk@aol.com; Tel.: +1-301-588-8875

**Keywords:** Drug use, substance abuse, sexual orientation, HIV risk behaviors, sexual risk practices, internet, men who have sex with men (MSM), content analysis

## Abstract

The present study entailed conducting a content analysis of 1,434 ads/profiles posted on one of the most popular “Men who have Sex with Men” (MSM) websites that specifically fosters unprotected sex. Ads/profiles were selected randomly based on the American ZIP code of residence (*n* = 1,316), with a randomly-drawn oversampling of profiles of men who self-identified as heterosexual or “curious” rather than gay or bisexual (*n* = 118). Data were collected between September 2006 and September 2007. The purpose of the present paper is to examine the conjoint effects of self-identified sexual orientation and preference for having/not having sex while high, on men’s sought-after sexual risk. Analytical comparisons of the four groups showed that, on most measures, the combination of sexual orientation and drug use preference during sex differentiated the men. Generally speaking, gay/bisexual men who advertised online for partners with whom they could have sex while high expressed the greatest interest in risky sexual behaviors (e.g., felching, unprotected oral sex, unprotected anal sex) and various risk-related preferences (e.g., multiple partner sex, anonymous sex, eroticizing ejaculatory fluids). This is especially true when they are compared to their heterosexual/“curious” counterparts whose online profiles were not as likely to indicate a desire for having sex while high.

## 1. Introduction

Men who have sex with other men (MSM) comprise the largest proportion of Americans who have been diagnosed with HIV or AIDS, accounting for 57% of all reported cases of AIDS with a known source of transmission and 57% of all HIV-positive persons who believed that they knew how they became HIV-infected [1]. Despite the so-called “changing face of AIDS in America,” these percentages for MSM have declined very little during the past ten years [2,3].

In light of this, numerous studies have been conducted to identify why, 25+ years into the HIV/AIDS epidemic, so many men continue to place themselves at risk for contracting HIV and/or other sexually transmitted infections. Many factors have been identified, including the belief that engaging in unprotected sex is an expression of individual choice [4,5], the belief that engaging in unprotected sex is an expression of masculinity [6–8], the perception that AIDS antiretroviral drugs have made HIV/AIDS less of a health concern now than in prior years [9,10], a fear of being rejected sexually by partners who dislike condoms [10], the belief that sex is more pleasurable when condoms are not used [11–13], feeling “burned out” by worrying about becoming HIV-infected [9,12], and feeling a greater sense of emotional connectedness to sexual partners with whom one had unprotected rather than protected sex [13,14].

How men who wish to have high-risk sex with other men locate potential sex partners has been the subject of relatively little research, however. For many men, “traditional” avenues of meeting other men–e.g., gay bars, gay/bisexual-oriented social activities, personal ads–remain popular ways of meeting potential sex partners. Another common way for men who wish to have unprotected sex with other men to locate potential partners is by frequenting public venues (e.g., parks, rest areas, rest rooms) where male-to-male cruising is known to take place. In recent years, with the proliferation of the internet, many men who wish to find other men specifically for engaging in unprotected sex appear to be turning to MSM-oriented websites for this purpose. For example, in a sample of gay men who were recruited into a health promotion study via gay-oriented internet websites [15], Bolding and colleagues’ multivariate analysis revealed that the amount of risky sex in which men engaged was a significant predictor of their use of internet websites to locate sex partners. In another study [16], among men actively using the internet as a means of locating potential sex partners, 97% reported actually having met someone online for sex, and 86% said that they used internet MSM sex sites at least once a week to identify possible partners. Another study examining the role that internet usage plays with regard to HIV risk taking found that persons who had a history of meeting sex partners via the internet reported more frequent involvement in risky sexual behaviors than persons who had not met sex partners online [17]. Comparable findings were reported by Benotsch, Kalichman and Cage [18], whose study of Atlanta area gay men found a greater likelihood of methamphetamine use, a larger number of sex partners, and a greater proportion of unprotected sex among men who used the internet to find sex partners. Similarly, in an online survey of men who engage in sex with other men, Berg [19] reported that those who use the internet to find sex partners were more likely than those who did not to engage in unprotected anal sex. Based on a multi-site internet study of MSM, Mutanski [17] found that a history of online sex-seeking was associated with a greater number of past-year sex partners, a larger number of one-time sex partners, more unprotected sex, and a lack of discussing sex partners’ sexual histories. Clearly, there has been mounting evidence of the importance of the role that the internet plays in fostering sexual encounters between men who specifically wish to have unprotected sex with other men.

Not as well understood, however, is the role that the internet plays in fostering drug use/abuse among MSM who meet for sex. This is, in part, the focus of the present paper. Research that *has* been done on this subject indicates a tendency for drug use to be greater or more problematic among men who use the internet to find sex partners. For example, Taylor, Aynalem, Smith, Montoya and Kerndt [20] reported that methamphetamine use was greater among men in their sample who used the internet to identify partners for sex. As another example, based on a comparison of men recruited from the internet and those recruited from a local Miami-area community, Fernandez and colleagues [21] found that club drug use was higher among MSM from their internet sample. They also noted that the club drug use in their internet sample was associated with a greater propensity to engage in unprotected sex–a finding that was not replicated among their non-internet-recruited study participants. Compounding the preceding problems, some research has shown that drug users are less inclined than those who do not use illegal drugs to participate in internet-based HIV risk reduction intervention programs [22]. As a general rule, however, more needs to be learned about the role that the internet plays in fostering drug use encounters and/or drug-related problems among MSM using websites to identify potential sex partners.

Also not well-understood is the role that the internet plays in fostering sexual encounters and risky practices among men who do not self-identify as gay or bisexual, but who use the internet to find other men with whom they can have sex. This is the other main focal area of the present study. Although behaviorally men who have sex with other men can be classified as gay or bisexual, in terms of their self-identity, not all of these persons consider themselves to be gay or bisexual. Indeed, many men who have sex with other men think of themselves as heterosexual/straight, or choose to label themselves as “sexually curious” instead. Both in the mass media and in the scholarly literature, these men are sometimes referred to as “being on the down-low” [23–25] or as engaging in “dude sex” [26]. Research on their sexual and risk behavior practices is sparse, but what *has* been published suggests that MSM who do not consider themselves to be gay or bisexual often engage in a variety of risky practices. For example, Siegel and colleagues [24] found that unprotected sex was common in this population, with nearly one-quarter of their study participants reporting both unprotected vaginal and unprotected anal sex during the preceding three months. Generally speaking, however, little is known about this population–a lament that has been voiced by other scholars as well [27,28].

Given: (1) the growing role that the internet appears to be playing in risky practices among MSM, (2) the general dearth of knowledge pertaining to drug use behaviors/problems, and (3) the dearth of information pertaining to risky practices among internet-using MSM who do not self-identify as gay or bisexual, the present study represents an effort to bridge part of this gap in knowledge. The emphasis in this paper is on sexual risk practices (both those entailing risk for HIV as well as those entailing risk for other sexually transmitted infections) and risk-related behavioral preferences sought by men who use the internet specifically to identify partners with whom they can engage in unprotected sex. Comparisons are made amongst four self-identified groups: gay/bisexual men who look online for partners with whom they can engage in sex while high, gay/bisexual men who look online for men with whom they can engage in sex while *not* being under the influence of alcohol or other drugs, heterosexual/“curious” men who use the internet to identify partners with whom they can engage in sex while high, and heterosexual/“curious” men who use the internet to find partners with whom they can engage in sex without being under the influence of alcohol or other drugs. Three main research questions are examined: First, are there differences in the extent to which men in these four groups seek risky behaviors in their online profiles? Second, do these four groups differ in terms of their risk-related preferences regarding how they want to have sex? Third, do the men in these four groups differ in terms of their sexual role preferences, their HIV serostatus, or the HIV serostatus they prefer in potential sex partners? Obtaining the answers to all of these questions is crucial if one wishes to understand how sexual orientation and drug use preference during sex mutually influence men’s behaviors, and then on that basis develop an informed prevention and/or intervention effort targeting the risk behaviors of men who use the internet to locate other men with whom they can engage in risky sex.

## 2. Methods

This research relies upon content analysis as the principal analytical tool. The data were collected between September 2006 and September 2007 using one of the largest MSM-oriented websites currently available on the internet. The website was chosen because it is free to the public, findable by virtually any internet search utilizing common key words like “bareback,” and because it boasts a large and steadily growing membership. At the time the data were collected, the site had more than 145,000 registered users (the large majority of whom resided in the United States) and it was growing at a rate of several hundred persons per month. This website allows members to post profiles (including photographs) describing themselves, and there are no length restrictions placed on profiles posted (see Endnote 1). In addition, there are specific places in their profiles where members are instructed to indicate the type(s) of relationships they are seeking (long-term relationships, one-on-one sexual encounters, three-way sexual encounters, and so forth), specific sexual acts that they would like to practice with a willing partner, and a free-for-all field that can be used to provide supplemental information about one’s most-sought-after traits or behaviors. Essentially, the large, stable, and growing membership of this website, coupled with members’ ability to describe themselves as fully as they choose, made this particular website an ideal candidate for the present content analysis research.

The content analysis was based on a random sample of users’ profiles, randomly selected by ZIP code, which is a searchable feature on the site. To facilitate comparisons based on sexual orientation, a random oversampling of 118 men who self-identified as heterosexual or “curious” supplemented the initial random sample (which also contained some men who considered themselves to be heterosexual or “curious”). Men residing outside of the United States were excluded from this research, so as to keep it an America-focused study. Also excluded from analysis (*n* = 6) were profiles that had not been filled out completely (i.e., with the user not providing at least one piece of the required information on each profile page on the website). In order to be included in the analyses, a user’s profile had to remain active at the conclusion of the data collection period, to guard against “experimenters” or one-time-only visitors to the site being included in the study. This led to the exclusion of 67 cases (4.8%). In all, 1,434 profiles constitute the sample for this research.

### 2.1. Data Collected

For each profile, the following information was collected: age; race/ethnicity (Caucasian, African American, Latino, Asian, Native American, or biracial/multiracial); self-identification as being a “top,” a versatile top, versatile, a versatile “bottom,” or a bottom (see Endnote 2); self-reported HIV serostatus (negative, positive, or unknown); desired HIV serostatus in sex partners (must be negative, may be negative, must be positive, may be positive, do not care); self-identified sexual orientation (gay, bisexual, “curious,” heterosexual); willingness to give and receive ejaculatory fluid in the mouth and anus; type(s) of “relationships” sought (one-on-one sexual encounter, long-term relationship, three-person sexual encounter, multiple partner sexual encounter, activities partner); the user’s ZIP code (which was used to compute population density as a macro-level analytical variable); and whether or not the user had opted for an expanded, paid membership on the site.

In addition, data collection also entailed coding for a wide variety of specific sexual behaviors, including among others felching (eating ejaculatory fluid that has been inserted into one person’s anus and then feeding it back to that individual by mouth, usually with a kiss), rimming (oral stimulation of the anus), bukkake (ejaculating directly onto another person’s mouth and face), and double penetration (forcing two penises into the same anus simultaneously). Finally, a variety of risk-enhancing practices and attitudes (hereinafter referred to as “risk preferences”) were also coded, including a stated preference for engaging in rough sex, having sexual relations while high (known in the target community as PNP, or “partying and playing”), overtly stating that they will not use condoms and/or that they will not permit their partners to use condoms, actively trying to become HIV-infected (known in the target community as “bug chasing”), actively trying to infect partners with HIV (known as “gift giving”), refusing to withdraw the penis prior to ejaculation and/or refusing to allow a sex partner to withdraw his penis prior to ejaculation, an overt preference for anonymous sex (i.e., sexual encounters in which the name and/or face of the sexual partner(s) is/are unknown), a stated preference for having long-lasting sexual encounters, an expression of seeking sexual encounters that are “uninhibited” or “no holds barred,” and eroticizing ejaculation fluid (known in the target community as being a “cum whore” or a “cum freak” or a “cum lover”).

The principal independent variable used in these analyses is a categorical measure combining information pertaining to men’s sexual orientation (self-identified, as described above) and their preference for/against having sex while high. Four groups are compared: (1) gay/bisexual men who look online for partners with whom they can engage in sex while high (*n* = 722), (2) gay/bisexual men who look online for men with whom they can engage in sex while *not* being under the influence of alcohol or other drugs (*n* = 578), (3) heterosexual/“curious” men who use the internet to identify partners with whom they can engage in sex while high (*n* = 44), and (4) heterosexual/“curious” men who use the internet to find partners with whom they can engage in sex without being under the influence of alcohol or other drugs (*n* = 90).

### 2.2. Research Questions and Analysis

Research Question #1 is “Are there differences in the extent to which men in these four groups seek risky behaviors in their online profiles?” Analytically, this can be examined with chi-square tests, since the dependent variables in question (e.g., felching, insertive/receptive oral sex, insertive/receptive anal sex) are dichotomous in nature. *Post hoc* comparisons tests were performed for all statistically-significant main effects relationships, to “tease out” the specific nature of the findings regarding sexual orientation versus drug use preference during sex versus the interaction effects of both of these measures. These *post hoc* tests entailed the computation of odds ratios, because this particular statistical method enables direct comparison of one group (or combination of subgroups) with another. Research Question #2 is “Do these four groups differ in terms of their risk-related preferences regarding how they want to have sex?” This too can be answered with chi-square tests (and *post hoc* testing as just described, whenever appropriate) because, once again, the dependent variables are dichotomous measures (e.g., wanting rough sex, seeking anonymous sex, wanting long-lasting sex). Research Question #3 is “Do the men in these four groups differ in terms of their sexual role preferences, their HIV serostatus, or the HIV serostatus they prefer in potential sex partners?” Again, because these measures are dichotomous or categorical in nature, chi-square tests (and *post hoc* testing as just described, whenever appropriate) are appropriate analytically. Results are reported as statistically-significant whenever *p <* 0.05.

## 3. Results

### 3.1. Sample

Men ranged in age from 18 to 64 (mean = 35.7, median = 36, *s.d.* = 8.8). The sample approximates the American population fairly well in terms of its racial composition, with 75.5% of the men being Caucasian, 9.0% African American, 7.6% Latino, 2.7% Asian, 0.4% Native American, and 5.0% biracial/multiracial. One-third of the men (35.4%) self-identified as being “top” or “versatile top”; one-quarter (22.4%) self-identified as being “versatile”; and the remainder (42.3%) self-identified as being a “bottom” or a “versatile bottom.” Most (62.6%) said that they were HIV-negative, although sizable proportions of the men whose ads were coded said that they knew that they were HIV-positive (16.0%) or that they did not know what their HIV serostatus was (21.4%). The sample, like the American population in general, tended to be skewed toward people residing in more-densely-populated areas. One-fifth of the men (19.7%) lived in an area with fewer than 250 persons per square mile. At the other end of the spectrum, 36.8% of the men resided in an area with more than 5,000 persons per square mile, and more than half of *these* men (19.5% of the total sample) lived in a high-density urban area with more than 10,000 persons per square mile.

### 3.2. Research Question #1: Differences in Sexual Risk Practices Sought, Based on Sexual Orientation and Drug Use Preference during Sex

For all of the sexual risk measures examined except one (the exception being insertive anal sex), significant differences were found in risk-seeking based on men’s sexual orientation and their preference for using/not using drugs during sex (see Table 1). A statistically-significant main effect was obtained for insertive oral sex involving internal ejaculation (χ^2^ [3df] = 13.39, *p =* 0.0039). *Post hoc* tests revealed that men who considered themselves to be heterosexual or “curious” and who did not want to have sex while high were the least likely of the four groups to want to find partners for receptive oral sex involving internal ejaculation (*OR* = 0.41, *CI_95_* = 0.24–0.71, *p* = 0.0004). A significant main effect was also obtained for receptive oral sex involving internal ejaculation (χ^2^ [3df] = 42.99, *p* < 0.0001). *Post hoc* analyses conducted here revealed that gay or bisexual men, regardless of their preference for/against having sex while high, were nearly three times as likely as the heterosexual or “curious” men in the sample to seek partners who would provide semen to them internally via oral sex (*OR* = 2.92, *CI_95_* = 1.99–4.27, *p <* 0.0001). As Table 1 shows, a statistically-significant main effect was obtained for receptive anal sex as well (χ^2^ [3df] = 49.33, *p* < 0.0001). Here, the *post hoc* analysis showed that, once again, gay and bisexual men, regardless of their preference for/against having sex while high, were more than twice as likely as their heterosexual or “curious” counterparts to want to find men who would perform anal sex on them and accept their semen anally (*OR* = 2.60, *CI_95_* = 1.79–3.78, *p <* 0.0001). They also revealed, though, that the gay and bisexual men who wanted to have sex while high were nearly twice as likely as gay/bisexual men who preferred not to have sex while high to want to find partners who would perform anal sex on them and ejaculate inside of them (*OR* = 1.73, *CI_95_* = 1.35–2.21, *p <* 0.0001). A statistically-significant main effect was also noted for men wanting to engage in all four of the preceding activities (i.e., receptive oral sex, receptive anal sex, insertive oral sex, and insertive anal sex) (χ^2^ [3df] = 42.27, *p* < 0.0001). Once again, the post hoc tests showed that gay and bisexual men, regardless of their preference for/against drug use during sex, were more likely than their heterosexual and “curious” counterparts to report wanting to engage in all four of these risky behaviors (*OR* = 2.79, *CI_95_* = 1.83–4.24, *p <* 0.0001). Moreover, gay/bisexual men who wanted to find partners for PNP were more likely than gay/bisexual men who did not want to find partners for PNP to say that they wanted to engage in receptive and insertive oral and anal sex (*OR* = 1.49, *CI_95_* = 1.19–1.87, *p =* 0.0004).

As Table 1 portrays, statistically-significant main effects were observed for the practices of felching (χ^2^ [3df] = 20.96, *p* < 0.0001) and rimming (χ^2^ [3df] = 13.03, *p =* 0.0046) as well. Regarding the former, gay/bisexual men, irrespective of their preference for/against finding partners with whom they could have sex while high, were nearly three times as likely as their heterosexual/curious counterparts to want to identify men with whom they could engage in felching (*OR* = 2.75, *CI_95_* = 1.33–5.88, *p =* 0.0029). *Post hoc* tests showed that, among the gay/bisexual men, it was those who preferred to have sex while high who were more likely to want to find partners for felching, particularly when compared to those who did not express a preference for having sex while high (*OR* = 1.69, *CI_95_* = 1.23–2.33, *p =* 0.0007). Similarly, rimming was sought significantly more often by the gay and bisexual men than it was by the heterosexual and “curious” men, regardless of their desire for/against having sex while high (*OR* = 3.25, *CI_95_* = 1.26–9.16, *p =* 0.0073). The *post hoc* analysis also revealed that gay/bisexual men who preferred to have sex while high were more likely than gay/bisexual men who preferred not to have sex while high to use the internet to advertise for partners with whom they could engage in rimming (*OR* = 1.49, *CI_95_* = 1.03–2.15, *p =* 0.0266).

### 3.3. Research Question #2: Differences in the Risk-Related Preferences, Based on Sexual Orientation and Drug Use Preference during Sex

Table 2 presents the findings obtained for the items pertaining to risk-related preferences. For three of the measures examined–wanting rough sex, overtly refusing to withdraw the penis prior to internal ejaculation (or allowing one’s partner to do so), and wanting long-lasting sex–no significant differences were found based on men’s sexual orientation and their preference for/against having sex while high. Intergroup differences were obtained for all of the other risk preference measures studied, though. For example, a significant main effect was noted for expressing an interest in multiple partner sex (χ^2^ [3df] = 40.73, *p <* 0.0001). *Post hoc* testing showed that gay/bisexual men, regardless of their PNP-related preference, were more likely than heterosexual/“curious” men to seek multiple partner sex online (*OR* = 2.62, *CI_95_* = 1.80–3.83, *p <* 0.0001). As another example, overtly expressing a dislike for condoms differed on the basis of men’s sexual orientation and their drug use preference during sex (χ^2^ [3df] = 13.42, *p =* 0.0038). Here, the *post hoc* comparisons revealed that the gay/bisexual men who wanted to engage in sex while high were significantly more likely than all other groups—about three times as likely overall—to state specifically in their profiles that they disliked condoms (*OR* = 3.25, *CI_95_* = 1.57–6.84, *p <* 0.0004). Additionally, posting a profile indicating an interest in anonymous sex differed on the basis of sexual orientation and preference for drug use during sex (χ^2^ [3df] = 9.71, *p =* 0.0212). Again, the *post hoc* comparisons revealed that the gay/bisexual men who wanted to engage in sex while high were significantly more likely than all other groups—nearly three times as likely overall—to state specifically in their profiles that they wanted to engage in anonymous sex (*OR* = 2.71, *CI_95_* = 1.33–5.60, *p =* 0.0026). Sexual orientation and drug use preference during sex were also associated with men’s likelihood of seeking “wild” or “uninhibited” sex via their online profiles (χ^2^ [3df] = 8.94, *p =* 0.0301). Yet again, the *post hoc* comparisons revealed that the gay/bisexual men who wanted to engage in sex while high were significantly more likely than all other groups to state in their profiles that they wanted to engage in “wild” or “uninhibited” sex (*OR* = 1.62, *CI_95_* = 1.04–2.54, *p =* 0.0242). As Table 2 shows, a significant main effect was obtained for eroticizing ejaculatory fluids (χ^2^ [3df] = 14.13, *p* = 0.0027). Closer examination of this relationship revealed that gay/bisexual men, regardless of their PNP-related preferences, were four times more likely than their heterosexual/“curious” counterparts to eroticize ejaculatory fluids (*OR* = 4.16, *CI_95_* = 1.74–10.59, *p =* 0.0003). A statistically-significant main effect was also derived for seeking sexual practices involving pain or harm to one or both partners (χ^2^ [3df] = 32.61, *p* < 0.0001). *Post hoc* analysis revealed that gay/bisexual men who wanted to find men with whom they could engage in sex while high were nearly twice as likely as all other groups of men to seek partners online for pain- or harm-involved sex (*OR* = 1.87, *CI_95_* = 1.48–2.37, *p <* 0.0001). Conversely, heterosexual/“curious” men who preferred having sex while high were significantly less likely than all other groups of men to seek partners for pain- or harm-involved sex (*OR* = 0.34, *CI_95_* = 0.13–0.86, *p =* 0.0116).

### 3.4. Research Question #3: Differences in Sexual Role Identity and HIV Serostatus Measures, Based on Sexual Orientation and Drug Use Preference during Sex

Table 3 provides a summary of the main measures of sexual role identity and HIV serostatus, as they pertain to men’s sexual orientation and their preference for being/not being high during sex. A significant main effect was observed regarding the proportion of men who considered themselves to be “tops” or “versatile tops” sexually (χ^2^ [3df] = 34.96, *p* < 0.0001). *Post hoc* comparisons tests demonstrated that heterosexual/“curious” men whose preference was not to have sex while high were more likely than all other groups of men to self-identify as sexual “tops” or “versatile tops” (*OR* = 2.67, *CI_95_* = 1.70–4.22, *p <* 0.0001), whereas gay/bisexual men who preferred to have sex while high were less likely than all other groups of men to consider themselves to be sexual “tops” or “versatile tops” (*OR* = 0.59, *CI_95_* = 0.47–0.74, *p <* 0.0001). A significant main effect was also obtained for the proportion of men who considered themselves to be “bottoms” or “versatile bottoms” sexually (χ^2^ [3df] = 24.22, *p* < 0.0001). *Post hoc* analysis showed that gay/bisexual men, regardless of their PNP-related preference, were more than twice as likely as their heterosexual/“curious” counterparts to identify themselves as “bottoms” or “versatile bottoms” (*OR* = 2.32, *CI_95_* = 1.52–3.55, *p <* 0.0001).

As Table 3 also depicts, sexual orientation and PNP-related preference were related to whether or not men were HIV-negative (χ^2^ [3df] = 65.74, *p* < 0.0001) or whether they said that they did not know what their current HIV serostatus was (χ^2^ [3df] = 40.50, *p* < 0.0001). *Post hoc* comparisons tests showed that heterosexual and “curious” men, regardless of their preference for/against having sex while high, were substantially more likely than their gay and bisexual counterparts to report being HIV-negative (*OR* = 8.56, *CI_95_* = 4.32–17.69, *p <* 0.0001). Conversely, irrespective of their PNP-related preferences, gay and bisexual men were substantially more likely than their heterosexual and “curious” counterparts to say that they did not know what their current HIV serostatus was (*OR* = 18.99, *CI_95_* = 11.99–30.43, *p <* 0.0001).

As Table 3 also shows, there were major intergroup differences based on desired sex partners’ HIV serostatus, based on men’s sexual orientation and their preference for/against having sex while high. A statistically-significant main effect was observed for stipulating that desired sex partners must be HIV-negative (χ^2^ [3df] = 95.57, *p* < 0.0001). *Post hoc* testing revealed that heterosexual/“curious” men, regardless of their PNP-related preference, were much more likely than their gay/bisexual counterparts to insist on finding HIV-negative sex partners (*OR* = 6.42, *CI_95_* = 4.13–10.01, *p <* 0.0001). A statistically-significant main effect was also obtained for profiles stating that the HIV serostatus of prospective respondents did not matter to the profile’s poster (χ^2^ [3df] = 79.04, *p* < 0.0001). *Post hoc* analysis demonstrated that gay and bisexual men, regardless of their PNP-related preference, were much more likely than their heterosexual or “curious” peers to say that they did not care about prospective partners’ HIV serostatus (*OR* = 5.63, *CI_95_* = 3.61–8.82, *p <* 0.0001).

## 4. Discussion

### 4.1. Potential Limitations of this Research

This content analysis research was conducted using one specific website and, therefore, there is no way to know whether users of this particular site are similar to or different from those who frequent other sites instead. Other sites were excluded from consideration in this research because of the fees that they charge in order for would-be users to partake of their services (see Endnote 3) and/or because of the significant limitations they placed on members’ ad/profile content. As a website specifically designed to foster contacts between men who wish to locate sexual partners with whom they can have unprotected sex, rather than being a website designed to foster male-to-male contacts of a more-general nature (e.g., dating, friendships, activities partners), the website chosen as the focus of this research represents an excellent sampling of men who are using the internet specifically to locate other men with whom they can have unprotected sex.

Another potential limitation of this research is that virtually all of the ads/profiles appearing on the website studied are written in English. Even though the website has a substantial Latino and multiracial membership (12.5% in total), fewer than 0.5% of the ads/profiles used a language other than English. This may prevent non-English speakers from utilizing the website, and this may limit the generalizability of the findings somewhat.

As a content analysis project, this research is unable to assess the extent to which the behaviors advertised for in the ads do or do not represent the behaviors practiced when people meet in person. For example, suppose someone has posted a profile stating that he does not care what his potential sex partner’s HIV serostatus is, and he meets an HIV-positive person who contacted him as a result of his profile. It is impossible to know whether the person would engage in all of the same sexual behaviors with a partner who is known to be HIV-positive versus a partner who is not HIV-positive or a partner with whom there is no discussion of HIV serostatus. In point of fact, the extent to which the risky behaviors advertised for and/or listed as preferences in the ads do or do not reflect actual behaviors practiced when people meet in person is, by necessity, the subject of a different study, following on the heels of the current project (see Endnote 4). Although some research has shown that men’s online profiles may, to some extent, be inaccurate [39], most published studies (cited earlier in this paper) showing that men who use the internet to locate sexual partners are very likely to meet up with such individuals for sex (i.e., their ads/profiles are, far more often than not, not posted purely for fun, but rather with sexual hook-ups in mind) suggest that there may not be a great disconnect between ad/profile content and behavioral practices. Nevertheless, this needs to be established with additional research.

Finally, the present author would like to acknowledge that some of the cell sizes for the analytical comparisons being made are smaller than what might be considered optimal from a statistical standpoint. Consequently, intergroup differences for some of the comparisons, particularly those involving the heterosexual/“curious” men who use the internet to identify partners with whom they can engage in sex while high (*n* = 44), may have yielded statistically-nonsignificant findings despite fairly-sizable magnitudes of difference between the groups. The extent to which this so-called Type II error may have affected the study findings cannot be assessed with the present data; but the author wishes to be “up front” about this potential limitation of the current study.

### 4.2. Conclusions

Despite these potential–and the present author would contend, minimal–limitations, the present research has much to offer in terms of helping to understand the content of ads posted on websites designed to foster unprotected sexual encounters among men seeking to have sex with other men. First and foremost, this research has shown that, on most dimensions, there seems to be an interaction effect between sexual orientation and drug use preference during sex. With few exceptions among the outcome measures examined in this research, gay or bisexual men who preferred to have sex while high comprised the group whose online profiles expressed the greatest interest in high-risk sexual behaviors and in the types of risk-related preferences that also heighten HIV risk even further. Conversely, men who self-identified as heterosexual or “curious” and whose online profiles did not indicate a desire to have sex while high constituted the group typically expressing the lowest level of interest in high-risk sex and risk-related preferences. Unmistakably, gay and bisexual men who prefer to use drugs in conjunction with their sexual activities constitute a risk group in need of targeted intervention if their HIV risk levels are to be reduced. Other authors have written about the heightened HIV-related risks that often accompany substance use among men who have sex with other men [29,30], including some theoretical work designed to help understand and explain the interrelationship of substance use, disinhibition, and HIV risk taking [31]. Some authors have taken this one step farther, by noting the additional heightening of risk that is attendant with this practice when it entails meeting men via the internet [18,29,32]. The general consensus among such studies, as well as the present research, is that the internet appears to be fostering a variety of sexually unsafe behaviors among MSM, and that MSM who like to have sex while under the influence of drugs are at an especially great risk for contracting or transmitting HIV and other sexually transmitted infections.

Another key finding obtained by the present research is that sexual role identity as a “top” or as a “bottom” differs significantly based on the combination of one’s sexual orientation and preference for/against drug use during sex. Gay and bisexual men who preferred to have sex while high were less likely than all other groups to consider themselves to be sexual “tops” and, conversely, more likely than all other groups to self-identify as “bottoms.” Moreover, men who considered themselves to be heterosexual or “curious” were the most likely to label themselves as sexual “tops.” These findings coincide with those discussed above for HIV risk practices and risk-related preferences, but extend those findings by addressing the issue of sexual role identity and its relationship to risk practices and preferences. By virtue of the sexual behaviors that they most desire, men who are “bottom”-oriented are seeking behaviors that are more likely to entail personal risk for contracting HIV and other STIs, particularly when compared to men whose sexual preferences are for behaviors that are best conceived as “top”-type behaviors. For many men, self-identification as a “top” or as a “bottom” has been shown to be related to other factors such as sensation seeking and psychological/psychosocial functioning [33], as well as to HIV serostatus [34]. In the context of the present study, identifying oneself as a sexual “top” or as a “bottom” was found to differ based on the combination of one’s sexual orientation and preference for/against drug use during sex. Since the majority of MSM appear to label themselves based on this notion of being a “top” or a “bottom” or “versatile” [33], additional research would be wise to learn more about how these labels affect sexual decision making, safety versus risk-related behavioral practices, and so forth.

Finally, in this study, the interaction of sexual orientation and drug use preference during sex was found to be related quite strongly to men’s HIV serostatus *and* to the HIV serostatus of the men they hoped to find online. MSM who self-identified as heterosexual or “curious” and whose online profiles did not express an interest in having sex while under the influence of drugs were far more likely than their gay/bisexual counterparts to be HIV-negative, particularly if those counterparts were looking for PNP or “party and play” opportunities. Conversely, members of the latter group was considerably more likely than the former to say that they did not know what their HIV serostatus was. Moreover, heterosexual/“curious” MSM whose profiles expressed a preference for sober sex were substantially more likely than their gay/bisexual counterparts whose profiles indicated a desire for sex while high to say that they wanted to identify sex partners who were HIV-negative, whereas the latter were much more apt than the former to say that they did not care about the HIV serostatus of potential sex partners. These particular findings highlight the importance of emphasizing both HIV testing and partner communication among men who have sex with other men, particularly if they are using the internet to identify potential sex partners. This is of especial importance with regard to the findings for being unaware of one’s own HIV serostatus and/or indifferent to the HIV serostatus of one’s partners, because numerous studies have shown lack of awareness of HIV serostatus to be associated with greater involvement in risky practices [35–37]. The present study’s finding suggests that future HIV intervention efforts working with MSM who use the internet need to target those who consider themselves to be gay or bisexual and who prefer to have sex while high, because these men are the ones who are least likely to be HIV-negative, the ones who are the least likely to insist on finding partners who are HIV-negative, and the ones who are the most likely to be unaware of their own and unconcerned about the HIV serostatus of their potential sex partners.

In conclusion, this content analysis study of men’s profiles on one of America’s largest unprotected sex-oriented websites has shown that there appears to be an interaction between sexual orientation and drug use preferences during sex. The combination of being gay or bisexual and wanting to have sex while under the influence of drugs appears to be associated with a variety of behaviors, risk preferences, and HIV testing-related attitudes that are conducive to the spread of HIV. Targeted intervention efforts are needed for members of this group, to help them deal with their substance use/abuse issues, to enhance their communication skills with their sex partners, and to encourage them to be tested for HIV on a regular basis.

## Figures and Tables

**Table 1.** Differences in Sexual Risk Practices Sought, by Sexual Orientation and Drug Use Preference.

**Sexual Risk Practice Sought**	**Gay/Bisexual, no drugs w/sex**	**Hetero/Curious, no drugs w/sex**	**Gay/Bisexual, drugs w/sex**	**Hetero/Curious, drugs w/sex**	**Statistical Significance**
Oral sex – receptive	74.4	53.3	81.0	59.1	*p* < 0.0001
Anal sex – receptive	63.3	44.4	74.9	52.3	*p* < 0.0001
Oral sex – insertive	87.2	75.6	89.1	88.6	*p* = 0.0039
Anal sex – insertive	80.5	70.0	79.4	81.8	*p* = 0.1462
All four of the preceding	44.1	21.1	54.0	36.4	*p* < 0.0001
Felching	12.6	6.7	19.7	6.8	*p* = 0.0001
Oral-anal contact	9.3	4.4	13.3	2.3	*p* = 0.0046

**Table 2.** Differences in Risk-Related Preferences, by Sexual Orientation and Drug Use Preference.

**Risk-Related Preference**	**Gay/Bisexual, no drugs w/sex**	**Hetero/Curious, no drugs w/sex**	**Gay/Bisexual, drugs w/sex**	**Hetero/Curious, drugs w/sex**	**Statistical Significance**
Wanting multiple partner sex	71.5	51.1	69.4	61.4	*p* < 0.0001
Wanting rough sex	12.1	8.9	11.2	6.8	*p* = 0.6176
Overtly stating dislike for condoms	1.7	0.0	4.9	2.3	*p* = 0.0038
Overtly refusing to withdraw the penis	1.7	0.0	2.1	2.3	*p* = 0.5682
Wanting anonymous sex	1.9	1.1	4.4	0.0	*p* = 0.0212
Wanting long-lasting sex	6.8	3.3	6.0	2.3	*p* = 0.4244
Seeking “wild” or “uninhibited” sex	6.1	2.2	8.2	0.0	*p* = 0.0301
Eroticizing ejaculatory fluids	15.4	3.3	17.0	6.8	*p* = 0.0027
Seeking sex involving pain/harm	25.6	21.1	37.5	13.6	*p* < 0.0001

**Table 3.** Differences in Sexual Role Identity and HIV Serostatus Measures, by Sexual Orientation and Drug Use Preference.

**Identity / Serostatus Measure**	**Gay/Bisexual, no drugs w/sex**	**Hetero/Curious, no drugs w/sex**	**Gay/Bisexual, drugs w/sex**	**Hetero/Curious, drugs w/sex**	**Statistical Significance**
Self-identifies as “top”	38.9	57.8	29.4	40.9	*p* < 0.0001
Self-identifies as “bottom”	40.0	25.6	47.2	25.0	*p* < 0.0001
HIV serostatus = HIV-negative	63.3	94.4	55.8	88.6	*p* < 0.0001
HIV serostatus = don’t know	19.7	1.2	26.2	2.5	*p* < 0.0001
Partner’s HIV serostatus = must be HIV-negative	37.7	78.9	33.0	75.0	*p* < 0.0001
Partner’s HIV serostatus = don’t care	58.3	21.1	62.9	22.7	*p* < 0.0001

## Footnotes

1 In contrast, most other MSM-oriented websites impose fairly-stringent limitations on the length of members’ postings, thereby precluding people from describing themselves and/or their sexual “want list” as completely as they might wish to do.

2 These terms refer to the sexual acts that the men typically prefer doing with their partners. For example, “top” men typically prefer to engage in insertive anal sex and to receive oral sex from their partners, whereas “bottom” men typically prefer to engage in receptive anal sex and to perform oral sex on their partners. These same “top” and “bottom” labels also may be applied to other sexual acts. Men who self-identify as “versatile” are usually willing to engage in sexual activities on a relatively even give-and-take basis.

3 This would have limited the generalizability of findings derived from studying their ad/profile content greatly, because socioeconomic factors such as the ability to afford site membership would have influenced site membership. With the site used in this research, membership is free to all who wish to utilize the website, and additional membership features are available for purchase for persons wishing to upgrade their usage of the site.

4 In fact, this very topic–the extent to which men’s online profiles do/not reflect their actual behaviors when they meet men from the internet–is the subject of a study currently being conducted by the present investigator. This study, which began in January 2008 and which concludes data collection during the spring of 2009, has shown that men’s online ads and profiles are an accurate representation of the things that they most like/want to do sexually. Of course, exceptions do exist; but overwhelming evidence from that study leads the present author to conclude that, by and large, men’s online ads and profiles depict the sexual behaviors that the men most want to practice when they consent to a face-to-face meeting with another man met online. Additional information about this project and some of its main findings may be found in Klein, Lambing, Moskowitz, Washington and Gilbert [38].
